# MicroRNA (let-7b-5p)-targeted DARS2 regulates lung adenocarcinoma growth by PI3K/AKT signaling pathway

**DOI:** 10.32604/or.2023.030293

**Published:** 2024-02-06

**Authors:** YUANYUAN XU, XIAOKE CHEN

**Affiliations:** Department of Oncology Surgery, Shanghai Lung Cancer Center, Shanghai Chest Hospital, Shanghai Jiao Tong University, Shanghai, China

**Keywords:** Lung adenocarcinoma, Prognosis, PI3K/AKT pathway, Mitochondrial aspartyl-tRNA synthetase, MicroRNAs

## Abstract

**Background:**

The aberrant intracellular expression of a mitochondrial aspartyl-tRNA synthetase 2 (DARS2) has been reported in human cancers. Nevertheless, its critical role and detailed mechanism in lung adenocarcinoma (LUAD) remain unexplored.

**Methods:**

Initially, The Cancer Genome Atlas (TCGA)-based Gene Expression Profiling Interactive Analysis (GEPIA) database (http://gepia.cancer-pku.cn/) was used to analyze the prognostic relevance of DARS2 expression in LUAD. Further, cell counting kit (CCK)-8, immunostaining, and transwell invasion assays in LUAD cell lines *in vitro*, as well as DARS2 silence on LUAD by tumorigenicity experiments *in vivo* in nude mice, were performed. Besides, we analyzed the expression levels of p-PI3K (phosphorylated-Phosphotylinosital3 kinase), PI3K, AKT (Protein Kinase B), p-AKT (phosphorylated-Protein Kinase B), PCNA (proliferating cell nuclear antigen), cleaved-caspase 3, E-cadherin, and N-cadherin proteins using the Western blot analysis.

**Results:**

LUAD tissues showed higher DARS2 expression compared to normal tissues. Upregulation of DARS2 could be related to Tumor-Node-Metastasis (TNM) stage, high lymph node metastasis, and inferior prognosis. DARS2 silence decreased the proliferation, migration, and invasion abilities of LUAD cells. In addition, the DARS2 downregulation decreased the PCNA and N-cadherin expression and increased cleaved-caspase 3 and E-cadherin expressions in LUAD cells, coupled with the inactivation of the PI3K/AKT signaling pathway. Moreover, DARS2 silence impaired the tumorigenicity of LUAD *in vivo*. Interestingly, let-7b-5p could recognize DARS2 through a complementary sequence. Mechanistically, the increased let-7b-5p expression attenuated the promo-oncogenic action of DARS2 during LUAD progression, which were inversely correlated to each other in the LUAD tissues.

**Conclusion:**

In summary, let-7b-5p downregulated DARS2 expression, regulating the progression of LUAD cells by the PI3K/AKT signaling pathway.

## Introduction

Lung cancer, a lethal malignancy arising from the respiratory epithelium, has emerged as one of the most lethal cancers, accounting for the enormous mortality of patients. According to 2020 statistics, a total of 228,820 newly estimated cases and 135,720 deaths were reported, accounting for 12.67% and 22.38% of all cancers, respectively [1]. Among various lung cancers, lung adenocarcinoma (LUAD) is the most common type, with an undesirable 5-year survival rate of around 18.0% [2]. The principal reasons could be insidious symptoms of LUAD and prognosis at the advanced stages, resulting in a deprived response to the treatment modalities. More often, the substantial treatment options for LUAD mainly rely on initial surgical resection followed by the systemic chemotherapeutic regimen [3]. Accordingly, there is an urgent need to identify critical biomarkers for early diagnosis and interpret their underlying mechanisms to reduce the disease burden.

To this end, the mitochondrial aspartyl-tRNA synthetase 2 (DARS2) is located in chromosome 1q25.1, containing 18 exons, resulting in the protein of the class-II aminoacyl-tRNA synthetase family. This mitochondrial enzyme plays a crucial role in the aminoacylation of aspartyl-tRNA explicitly. Oftentimes, mutations in DARS2 result in leukoencephalopathy with brainstem and spinal cord involvement and lactate elevation [4,5]. Previous reports highlighted the critical role of DARS2 in cancer progression. For instance, DARS2 upregulation was correlated with the dismal clinical outcome of patients with hepatocarcinogenesis, in which DARS2 was represented as an anti-oncogene by the mitogen-activated protein kinase (MAPK) pathway [6]. In another instance, the highly expressed DARS2 was prognostically worse in bladder cancer patients [7]. Despite the availability of some reports, the substantial role of DARS2 in LUAD remains incomprehensible.

The micro-ribose nucleic acids (miRNAs), referred to as hairpin-shaped single-stranded RNAs, are non-coding transcripts with approximately 20 nucleotides, functioning as important post-transcriptional regulators of gene expression. These miRNAs often act as imperfect sequence guides and directly repress target genes, similar to endogenous or synthetic short-interfering RNAs (siRNAs) [8]. Therefore, miRNAs play crucial roles in cellular communication, contributing to various cellular processes. Previous reports demonstrated that the abnormal expression of miRNAs exerted the oncogenic or tumor-suppressive functions in LUAD malignancy by interfering with mRNA expression [9]. In an instance, miRNA-181 suppressed chromobox 7 (CBX7) and reactivated the Wnt/β-catenin pathway, suggesting its oncogenic contribution to the LUAD progression [10]. In another instance, miRNA-301a-3p showed a complementary sequence with 3'UTR SNX2 and subsequently downregulated the anti-tumorigenicity of SNX2 in the LUAD progression [11]. In addition, several reports indicated that let-7b-5p acted as an oncological miRNA or tumor suppressor by regulating the targeted gene expression context dependently [12,13]. In an instance, a recent global profiling of miRNA identified the downregulation of let-7b-5p in the LUAD progression [14]. However, the comprehensive role, along with its underlying mechanism, remains unraveled.

Motivated by these facts and considerations, this study demonstrated the role of DARS2 expression in The Cancer Genome Atlas (TCGA)-LUAD cohort through the Gene Expression Profiling Interactive Analysis (GEPIA) database. Further, the integrative analysis of miRNA and mRNA microarray suggested let-7b-5p targeting DARS2. It was initially hypothesized that let-7b-5p interfered with DARS2 expression and constrained LUAD progression. Subsequently, we validated the functional attributes of DARS2 during LUAD progression through cell counting kit (CCK)-8, immunostaining, and transwell invasion assays in LUAD cell lines *in vitro*, and DARS2 silence on LUAD by tumorigenicity experiments *in vivo* in nude mice. Finally, the underlying mechanism behind let-7b-5p/DARS2 axis functioning in LUAD was analyzed.

## Methods

### Clinical samples

LUAD and adjacent normal tissues (*n* = 90 pairs) were harvested from patients who underwent their first tumor resection in Shanghai Chest Hospital (Shanghai, China) from January 2018 to April 2019. The collected specimens were sent for pathologic confirmation before performing the notified assays. It should be noted that written informed consent from each patient was obtained for this study. The study protocol complied with the ethical guidelines of the 1975 Declaration of Helsinki as reflected in prior approval by the Ethics Committee of Shanghai Chest Hospital.

### Cell culture and transfection

Human lung cancer cells (PC9 and A549 cell lines) and human bronchial epithelial cells (BEAS-2B cell line) were obtained from the American Type Culture Collection (ATCC, Manassas, VA, USA) and cultured in the standard Roswell Park Memorial Institute (RPMI)-1640 medium (ThermoFisher Scientific, Waltham, MA, USA) containing 10% fetal bovine serum (FBS, Invitrogen, Waltham, MA, USA) and 1% penicillin/streptomycin (ThermoFisher Scientific) and maintained in a CO_2_ cell culture incubator (5% CO_2_, 37°C).

The short-hairpin RNAs (shRNAs) targeting DARS2 (sh-DARS2 #1/2/3) and sh-NC were purchased from Gema (Shanghai, China). The pcDNA-DARS2 vectors (DARS2 vectors) and the empty vectors were obtained from GeneChem Co. Ltd. (Shanghai, China). These recombinant vectors were transfected into PC9 and A549 cell lines, seeded at a density of 1 × 10^5^ cells, via Lipofectamine 2000 (Lipo 2000, ThermoFisher, Waltham, USA). Further, after the required incubation period, the transfection efficiency was evaluated by reverse transcription-quantitative real-time polymerase chain reaction (RT-qPCR).

### RT-qPCR analysis

Initially, the TRIzol reagent (Sigma, St. Loius, USA) was used to extract total RNA from the LUAD cells and tissues. Further, the reverse transcription was undertaken using the PrimeScript™ 1^st^ Strand cDNA Synthesis Kit (TaKaRa Co. Ltd., Kusatsu, Japan). Then, the RT-qPCR analysis was performed on the HT7900 system (ABI system) using SYBR Green Real-Time PCR Master Mix (TaKaRa Co. Ltd.). Finally, the comparative 2^−ΔΔCT^ method was used to normalize the data against glyceraldehyde-3-phosphate dehydrogenase (GAPDH or U6).

The primers are listed as follows:

let-7b-5p: Forward-CAGTGAGGTAGTAGGTTGTGT

Reverse-CTCAACTGGTGTCGTGGA

U6: Forward-CTCGCTTCGGCAGCACA

Reverse-AACGCTTCACGAATTTGCGT

DARS2: Forward-CCCAAGAGGAAGATGTGGTCC

Reverse-AGAACAGAGTGGGGTCACG

GAPDH: Forward-CCTGCACCACCAACTGCTTAG

Reverse-GTGGATGCAGGGATGATGTTC

### Western blot analysis

Initially, PC9 and A549 cells were treated with radioimmunoprecipitation assay (RIPA buffer, Beyotime, Shanghai, China) and quantitatively assessed by a bicinchoninic acid (BCA) protein detection kit (Beyotime, Shanghai, China). 10 µg of the extracted protein was loaded on 15% sodium dodecyl sulfate-polyacrylamide gel electrophoresis (SDS-PAGE, Sigma-Aldrich) and subjected to separation before being transferred onto polyvinylidene fluoride (PVDF) membranes. Further, 2.5% of skimmed milk was used to block the protein-blotted PVDF membranes. Afterward, the membranes were incubated with primary antibodies against DARS2 (1:1000), GAPDH (1:1000), AKT protein kinase B, (p-AKT 1:1000), p-AKT (1:1000), AKT (1:1000), phosphorylated-Phosphotylinosital 3 kinase (p-PI3K, 1:1000), and PI3K (1:1000) obtained from Abcam (Cambridge, USA). After incubation overnight, the horse-radish peroxidase (HRP)-conjugated secondary antibodies (1:1000, Abcam) were used to detect the protein blots. Finally, the protein signals were visualized through Clarity Western electrochemiluminescence (ECL) substrate (Bio-Rad, Hercules, CA, USA).

### CCK-8 assay

A549 and PC9 cell lines at a density of 1 × 10^4^ cells per well were seeded in the 96-well plates and incubated for 24, 48, and 72 h. Afterward, 10 µL of CCK-8 reagent (Beyotime) was added to each well and incubated for 2 h. Finally, the plates were colorimetrically read on a microplate reader (ThermoFisher Scientific) at 450 nm, and the relative growth rate of the cells was calculated.

### EDU assay

A 5-ethynyl-29-deoxyuridine (EdU) assay kit (RiboBio, Guangzhou, China) was applied to detect the DNA synthesis in A549 and PC9 cell lines. 20 µl of EDU reagent was supplemented in the 24-well plates containing 1 × 10^5^ LUAD cells per well. After 3h, 4′,6-diamidino-2-phenylindole (DAPI) was consecutively added at room temperature for 30 min to counterstain the nuclei. Finally, the EdU-positive and DAPI-positive nuclei were observed under a fluorescence microscope (TheremoFisher).

### Flow cytometry

An annexin V-fluorescein isothiocyanate (FITC)/Propidium iodide (PI) apoptosis kit (ThermoFisher) was used to detect the apoptosis of LUAD cells. Initially, the LUAD cells at 85% confluence were treated with ethylene diamine tetraacetic acid (EDTA)-free trypsin and re-suspended in 100 μL of 1× binding buffer. Further, 5 μL of annexin V-FITC and 10 μL of PI staining mixture were added and kept in the dark at room temperature for 10 min. 400 μL of 1× binding buffer was added before apoptosis detection with a FACS Calibur Flow Cytometer (BD Biosciences, San Jose, USA).

### Transwell invasion assay

For the transwell invasion assay, the matrigel-coated inserts were implanted in the 24-well culture plates to assemble the invasion chamber. Further, a 100 μL medium suspended with 1 × 10^5^ A549 or PC9 cells was added to the upper chamber, and a 500 μL medium was supplemented with FBS in the lower chamber. After 36 h of incubation, the inserts were fixed using 4% paraformaldehyde (500 μL for 15 min) and counterstained in 500 μL of crystal-violet solution for another 15 min. Finally, the invaded cells were counted.

### In vivo investigations

Nude mice (*n* = 10) were purchased from the Experimental Animal Center of Wuhan University (Wuhan, China). Further, the nude mice were maintained under specific pathogen-free (SPF) conditions with food and water available *ad libitum*. After one week of adaptive feeding, nude mice received subcutaneous injection with A549 transfected with sh-DARS2 or sh-NC. After 35 days, the nude mice were sacrificed by treating them with CO_2_. Finally, the resected tumors were weighed and subjected to immunohistochemical (IHC) analysis. The protocol was proved by the Institutional Animal Care and Ethics Committee of Shanghai Chest Hospital.

### Immunocytochemistry (IHC) analysis

The collected tumor tissues from sacrificed mice were initially fixed with 4% paraformaldehyde, and wax blocks were prepared. Further, these blocks were sectioned in the thickness range of 2.5 μm. After permeabilizing with 0.1% Triton X-100 in PBS for 10 min, the thin tissue sections were subjected to primary antibodies against Ki67, DARS2, Cleaved-caspase 3, E-cadherin, and N-cadherin at 4°C. After 48 h, the Alexa Fluor 488 secondary antibodies (ThermoFisher Scientific) were used to detect the positive signals at room temperature for 30 min. After gradient alcohol dehydration, the sections were observed under the microscope. Finally, the IHC images were analyzed with optical density (OD) value by ImageJ software v1.41 (National Institutes of Health, Bethesda, USA).

### Luciferase reporter assay

The dual-luciferase reporter assay was performed as stated in the following procedure. Initially, the wild-type (WT) or mutant (MUT) binding sequence of let-7b-5p to DARS2 3′UTR was amplified and inserted into luciferase reporter vectors (Promega, Madison, WI, USA). Further, the constructed luciferase reporter vectors were coupled with let-7b-5p to mimic or mimic NC into A549 and PC9 cells. After 48 h, luciferase reporter systems were used to detect the luciferase activity following the manufacturer’s instructions.

### Chip analysis

GEPIA database was used to analyze the expression and the prognostic relevance of DARS2 in LUAD tissues. “miRDB”, “starBase”, “TargetScan”, and “Tarbase” were used to analyze the miRNAs targeting DARS2 3′UTR. The KM Plotter (www.kmplot.com) was used to assess the prognostic value of DARS2 in the LUAD tissues of the patients.

### Statistical analysis

The data were expressed in terms of mean ± standard deviation (S.D.) and processed using GraphPad Prism 9.0. In addition, Pearson’s analysis was conducted to analyze the relationship between DARS2 and let-7b-5p. The difference between the groups was analyzed using a student’s *t*-test for two groups or one-way ANOVA analyses for multiple groups, considering *p* < 0.05 as a statistically significant difference. * represents *p* < 0.05, ** defines *p* < 0.01, and *** indicates *p* < 0.001.

## Results

### DARS2 overexpression relates to the dismal clinical outcome of LUAD patients

**Figure 1:**
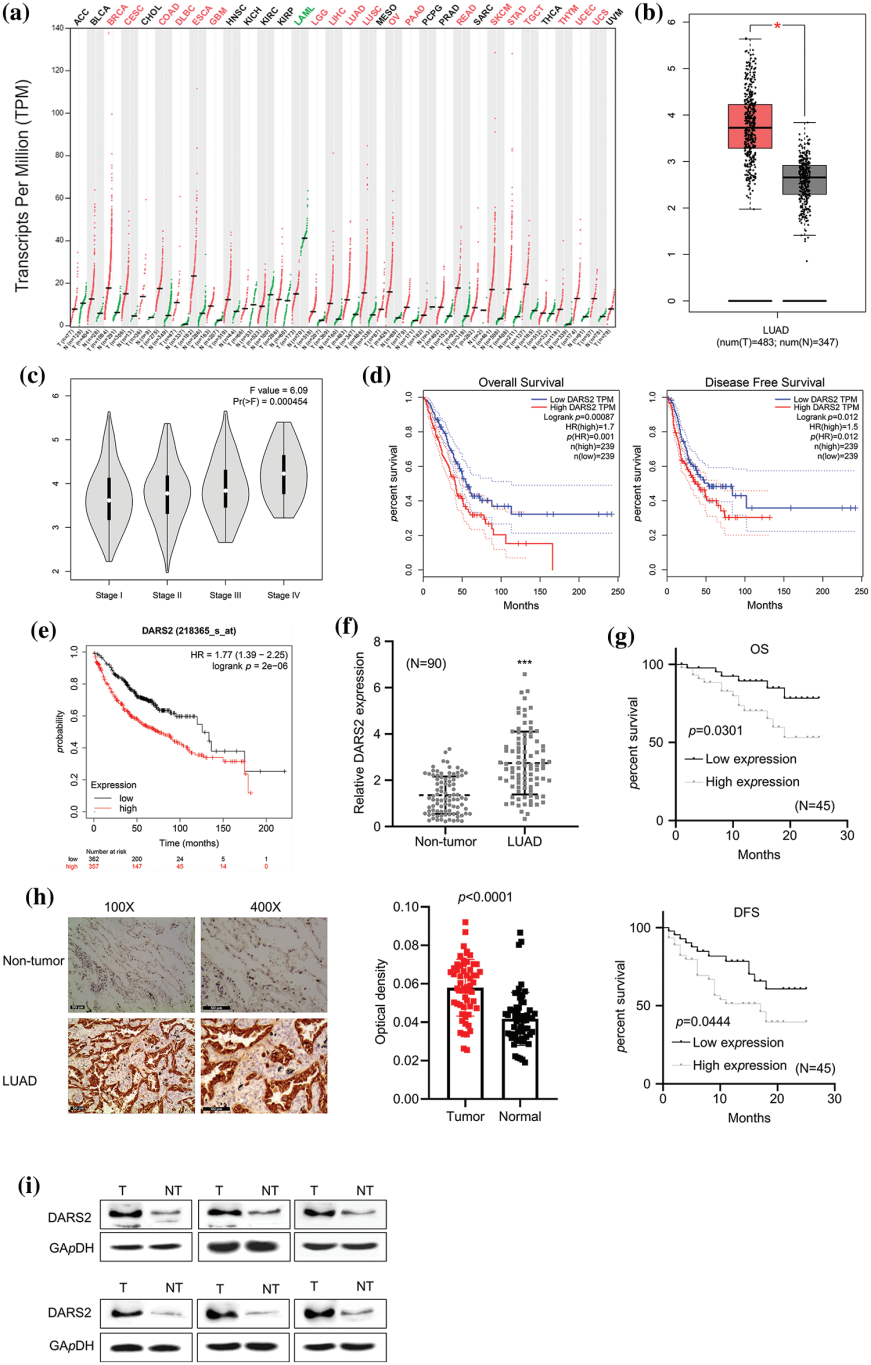
Overexpression of DARS2 is related to the dismal clinical outcome of LUAD patients. (a) Data mining in GEPIA shows the differential expression of DARS2 in pan-cancer. (b) GEPIA database analyzes DARS2 in LUAD tissues and normal tissues. (c) The graph represents the association between DARS2 expressions in LUAD with the corresponding stages in the GEPIA database. (d) GEPIA shows the overall survival and the disease-free survival of LUAD patients. (e) An online KM plotter indicates a survival analysis of DARS2 mRNA expression during LUAD malignancy. (f) RT-qPCR analysis shows DARS2 expression in LUAD tissues and normal tissues. (g) KM plotter and log-rank test compare the survival rate of DARS2-high and DARS2-low groups. (h) IHC analysis presents DARS2 protein expression in LUAD tissues and normal tissues. (i) Western blot analysis indicates DARS2 protein expression in LUAD tissues and normal tissues. *** indicates *p* < 0.001.

To explore the relationship between DARS2 overexpression and the clinical outcome in LUAD patients, we first determined the DARS2 mRNA expression in LUAD tissues from patients using RT-qPCR analysis. Further, the GEPIA database was employed. It was observed that DARS2 was differentially expressed in various cancerous tissues (Fig. 1a). Moreover, DARS2 expression was amplified in the LUAD tissues (*n* = 483) compared with the paracancerous tissues (*n* = 347) (Fig. 1b). In addition, the DARS2 mRNA levels were elevated from stage I to stage III (Fig. 1c). According to the GEPIA database results, the LUAD patients with high-DARS2 expression showed a worse overall survival rate and a shorter disease-free survival rate (Fig. 1d). Further, the KM Plotter (www.kmplot.com) was used to stratify the LUAD patient cohorts based on the median value of DARS2 and its evaluated prognostic significance. The highly expressed DARS2 was consistently associated with an unfavorable overall survival rate (Fig. 1e). To further validate the data from the bioinformatics analysis, LUAD tissues (*n* = 90 pairs) were collected and analyzed their survival information. RT-qPCR analysis results indicated that the expression levels of DARS2 in LUAD tissues were higher than in the adjacent normal tissues (Fig. 1f). Moreover, the survival analysis also validated the association of amplified DARS2 with a tendency toward prognostic pessimism in LUAD patients (Fig. 1g). Although there was no association with age, tumor size, and sex, the clinicopathological analysis by chi-square test indicated that DARS2 expression was associated with the Tumor-Node-Metastasis (TNM) stage and high lymph node metastasis (*p* values of 0.0082 and 0.0147, respectively, Table 1). Further, the IHC analysis presented higher DARS2 expression in tumors than in normal tissues (Fig. 1h). Similarly, the Western blot analysis also validated the amplification of DARS2 protein expression in the LUAD tissues (Fig. 1i). Together, these findings suggested that DARS2 could be critically involved in the LUAD malignancy.

**Table 1:** A summary presents the correlation between DARS2 expression and clinicopathological features of LUAD patients

Characteristics	n	DARS2 expression		*p*
		Low (*n* = 45)	High (*n* = 45)	
Age (years)				0.3894
<56	36	20	16	
≥56	54	25	29	
Gender				0.6121
Male	70	36	34	
Female	20	9	11	
Tumor size (cm)				0.0575
<3	47	28	19	
≥3	43	17	26	
Smoking				0.1337
Smokers	53	23	30	
Nonsmokers	37	22	15	
Tumor number				0.399
Single	46	21	25	
Multiple	44	24	20	
Stage				0.0082
I + II	58	35	23	
III + IV	32	10	22	
Lymph node metastasis				0.0147
Yes	31	10	21	
No	59	35	24	
Distant metastasis				0.081
Yes	14	4	10	
No	76	41	35	

### DARS2 silence constrains LUAD cell proliferation, migration, and invasion

**Figure 2:**
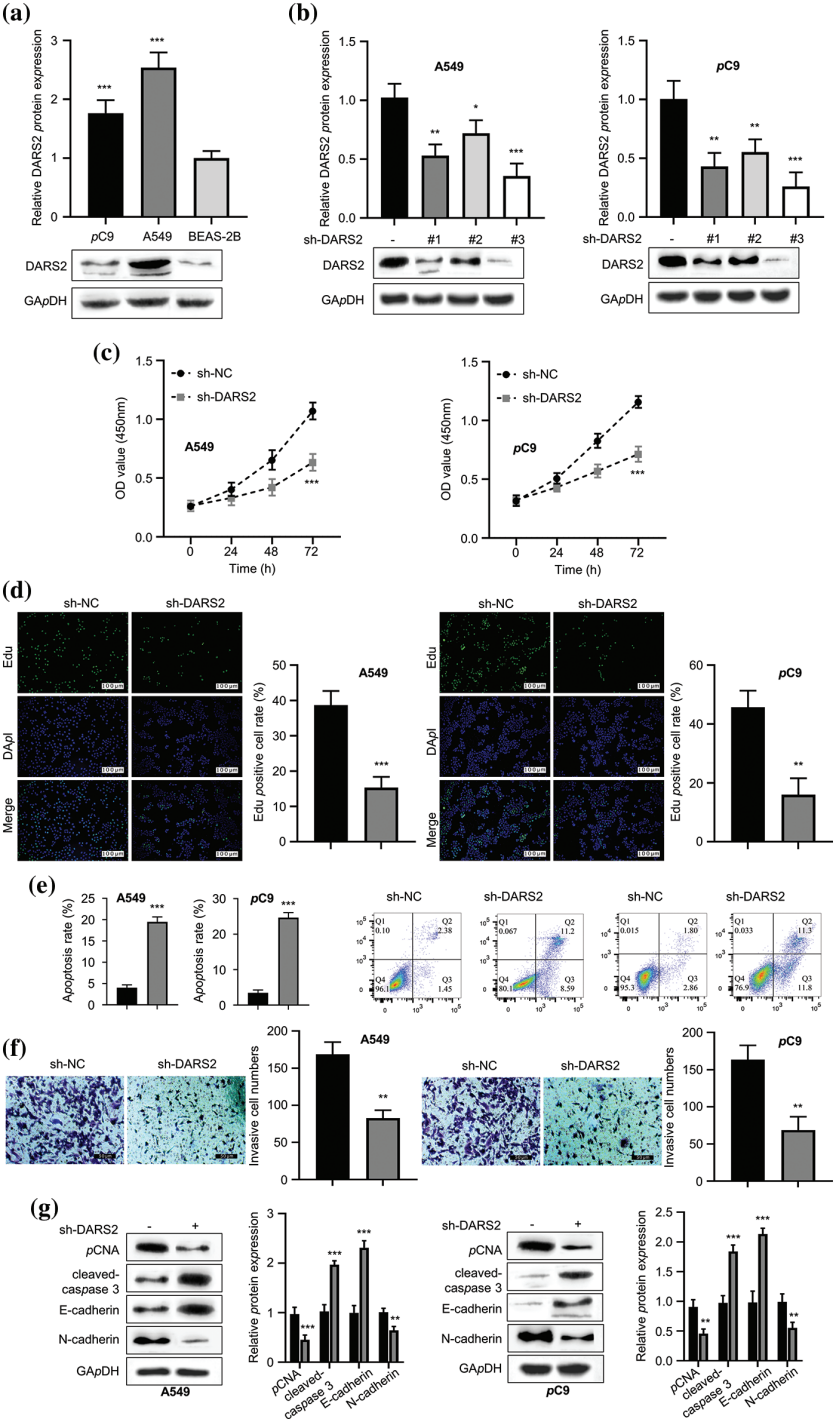
DARS2 silence constrains LUAD cell proliferation, migration, and invasion abilities. (a) Western blot analyzes the DARS2 expression in A549, PC9, and BEAS-2B cell lines. (b) Western blot analysis indicates the DARS2 expression in A549 and PC9 transfected with sh-NC and sh-DARS2 (sh#1, sh#2, and sh#3). (c) CCK-8 assay presents the LUAD cell proliferation in DARS2 silencing. (d) EdU assay detects LUAD cell proliferation, in which EdU stains red and DAPI stains blue. (e) Flow cytometry analysis demonstrates the LUAD cell apoptosis. (f) Transwell invasion assays evaluate the LUAD cell invasion. (g) Western blot analyzes the expression of PCNA, cleaved-caspase, PCNA, and N-cadherin proteins in LUAD cells. * represents *p* < 0.05, ** defines *p* < 0.01, and *** indicates *p* < 0.001.

Considering the theoretical evaluations, the relationship between the DARS2 expression and LUAD was validated *in vitro* in various LUAD cell lines and compared with normal cell lines. Further, the robust expression levels of DARS2 were observed in LUAD cells, i.e., PC9 and A549 cells, and compared concerning that of the normal lung epithelial cells, i.e., BEAS-2B cells (Fig. 2a). It was observed that the differentially expressed DARS2 implicated its contribution to the LUAD progression. To disclose the possibility, sh-DARS2, and sh-NC were delivered into LUAD cells (PC9 and A549 cell lines). The highly efficient DARS2 silencing was evidenced by its significant downregulation in PC9 and A549 cells after being transfected with sh-#1, sh-#2, and sh-#3 (Fig. 2b). We chose sh-#3 for the following biofunctional assays according to the silencing efficiency. It was observed that the DARS2 silencing substantially caused a significant reduction in the proliferation of PC9 and A549 cells (Fig. 2c). In addition, the EdU staining results indicated that DARS2 silencing reduced the proliferation of PC9 and A549 cells (Fig. 2d). As a result, a noticeable increase in the cellular apoptosis of PC9 and A549 cell lines was observed in the case of DARS2 silencing (Fig. 2e). A low expression of DARS2 also contributed to a decreased invasive rate of the selected two LUAD cell lines (Fig. 2f). To further corroborate the impact of DARS2 and its silencing on the proliferation and invasive phenotypes of LUAD cells, Western blot was employed to assess the expression levels of the proliferating protein markers (PCNA and cleaved-caspase-3) and migratory protein markers (E-cadherin and N-cadherin). It was observed that DARS2 silencing resulted in enhanced expression levels of cleaved-caspase-3 and E-cadherin proteins, while the expression levels of N-cadherin and PCNA proteins were reduced (Fig. 2g). Thus, these findings suggested that DARS2 silencing could substantially prevent LUAD cell proliferation and invasion *in vitro*.

### let-7b-5p targets DARS2

**Figure 3:**
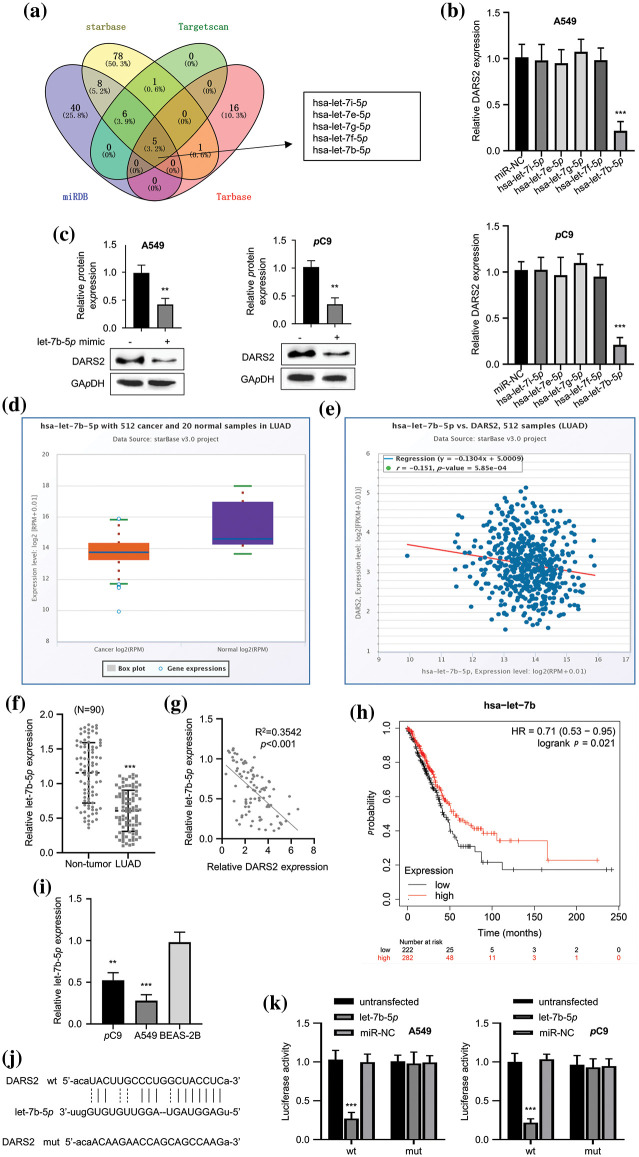
The let-7b-5p targets DARS2. (a) The online tools “miRDB”, “starBase”, “TargetScan” and “Tarbase” predicts the intersection of miRNAs targeting DARS2 mRNA 3′ UTR. (b) RT-qPCR analysis presents the DARS2 mRNA levels in A549 and PC9 cells while overexpressing hsa-let-7i-5p, hsa-let-7e-5p, hsa-let-7g-5p, hsa-let-7f-5p, and hsa-let-7b-5p. (c) Western blot analyzes the DARS2 mRNA levels in A549 and PC9 cells transfected with let-7b-5p mimic or mimic NC. (d) The starBase predicts the let-7b-5p expression in the TCGA-LUAD cohort. (e) The image indicates the correlation between the expression of hsa-let-7b-5p and DARS2 based on the TCGA-LUAD cohort. (f) RT-qPCR indicates the let-7b-5p expression in LUAD and normal tissues from 90 LUAD patients. (g) Pearson’s analysis demonstrates a correlation between the expression of hsa-let-7b-5p and DARS2 in LUAD tissues from 90 LUAD patients. (h) The KM Plotter (www.kmplot.com) displays the overall survival of 90 LUAD patients. (i) RT-qPCR analysis shows the let-7b-5p expression in LUAD cells (PC9 and A549) and normal human bronchial epithelial cell line BEAS-2B. (j) The image indicates the DARS2 3′UTR docking site with hsa-let-7b-5p. (k) The graph presents the luciferase activity driven by DARS2 3′UTR MUT or WT in A549 and PC9 cells transfected miR-NC, let-7b-5p or not. * represents *p* < 0.05, ** defines *p* < 0.01, and *** indicates *p* < 0.001.

miRNAs often recognize the 3′-untranslated region (3′-UTR) of mRNA and thereby downregulate the mRNA expression. To validate the mechanism behind the DARS2-driven LUAD progression, the upstream regulatory mechanism was first observed. To identify the target sites, several miRNA target site prediction tools, including “miRDB”, “starBase”, “TargetScan”, and “Tarbase”, were employed. Among the analyzed miRNAs, five miRNAs, i.e., hsa-let-7i-5p, hsa-let-7e-5p, hsa-let-7g-5p, hsa-let-7f-5p, and hsa-let-7b-5p were predicted as the intersections of miRNA to DARS2 (Fig. 3a). Nevertheless, substantial change in the DARS2 expression in the LUAD cells only after transfection with hsa-let-7b-5p (Fig. 3b). Moreover, the let-7b-5p mimic transfection also reduced the expression levels of DARS2 protein in LUAD cell lines (Fig. 3c). The hsa-let-7b-5p was poorly expressed in the LUAD tissues compared to tumor-free tissues (Fig. 3d). Interestingly, the GEPIA database demonstrated a negative correlation between hsa-let-7b-5p and DARS2 (Fig. 3e). The KM Plotter (www.kmplot.com) indicated that the poor expression of hsa-let-7b-5p was related to the worsening of the overall survival rate (Fig. 3f). In the clinical samples, the downregulation of hsa-let-7b-5p and its negative correlation with DARS2 were also detected in the LUAD cohort (Figs. 3g and 3h). Further, RT-qPCR results indicated that LUAD cells (A549 and PC9) showed lower expression levels of hsa-let-7b-5p compared with BEAS-2B (Fig. 3i). As predicted, hsa-let-7b-5p shared the complementary sequence with DARS2 mRNA 3′ UTR (Fig. 3j). The luciferase reporter assay showed the targeted relationship between hsa-let-7b-5p and DARS2, displaying that let-7b-5p mimic impaired the DARS2-WT-mediated luciferase activity while showing no effect on DARS2-MUT-mediated luciferase activity (Fig. 3k). Therefore, these results suggested that hsa-let-7b-5p targeted DARS2 3′UTR to interfere with DARS2 expression.

### DARS2 overexpression reverses the effects of let-7b-5p in LUAD cells

**Figure 4:**
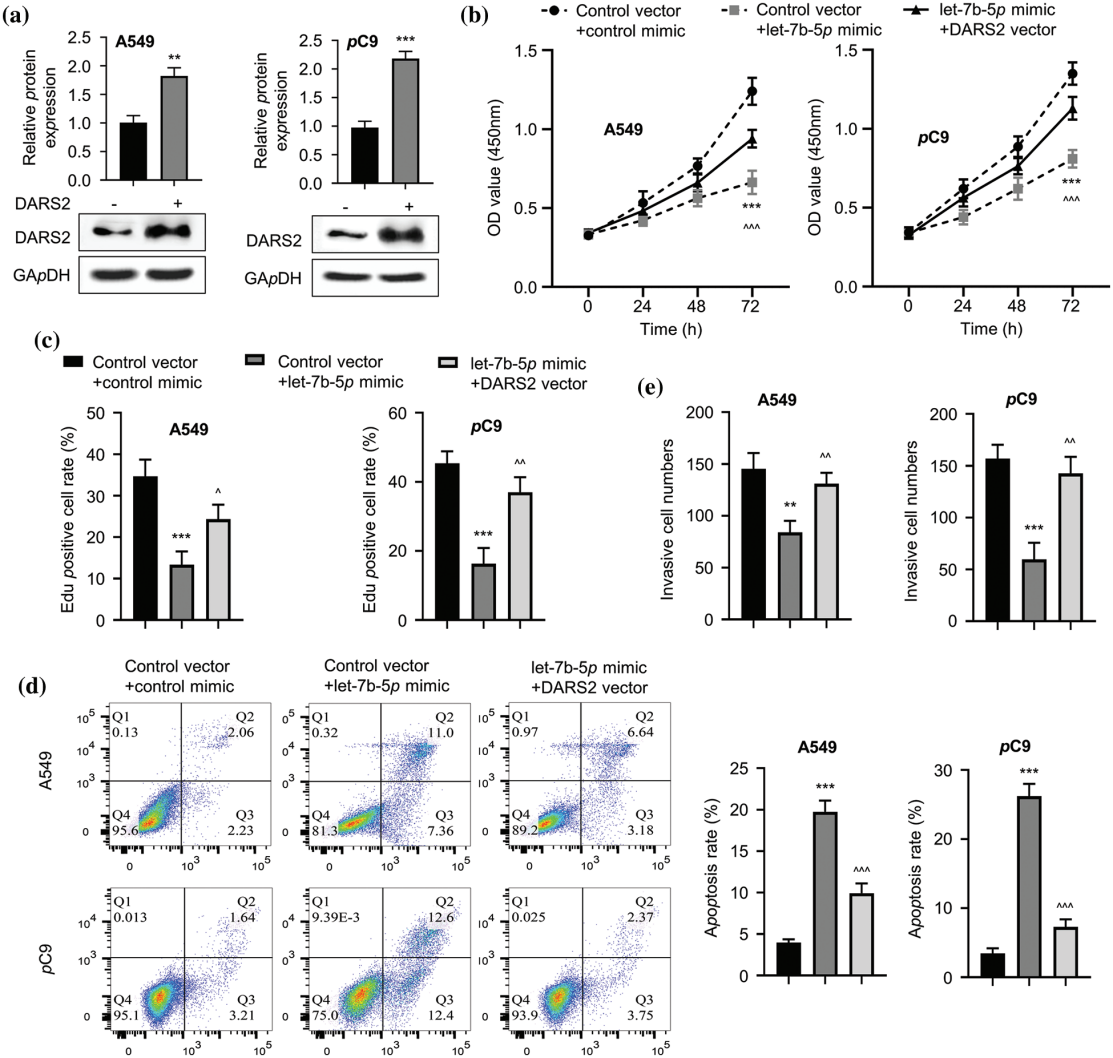
The DARS2 overexpression reverses the effects of let-7b-5p in LUAD cells. (a) Western blot analyzes the DARS2 expression in A549 and PC9 cells while DARS2 overexpression. (b) CCK-8 assay determines the proliferation of LUAD cells while DARS2 and let-7b-5p overexpression in A549 and PC9 cells. (c) EdU assay presents the LUAD cell proliferation in the case of DARS2 and let-7b-5p overexpression in A549 and PC9 cells. (d) Flow cytometric analysis detects apoptosis while DARS2 and let-7b-5p overexpression in A549 and PC9 cells. (e) Transwell invasion assessment presents cell invasion while DARS2 and let-7b-5p overexpression in A549 and PC9 cells. * represents *p* < 0.05, ** defines *p* < 0.01, and *** indicates *p* < 0.001.

To determine the impact of let-7b-5p on the action of DARS2, we overexpressed DARS2 in the LUAD cell lines (Fig. 4a) and then transfected with let-7b-5p mimic. As detected by CCK-8 and EDU assays, let-7b-5p mimic treatment considerably reduced the LUAD cell proliferation, which was further reinstated with DARS2 upregulation (Figs. 4b and 4c). Furthermore, let-7b-5p mimic treatment triggered the apoptosis activation, and additional DARS2 overexpression partially abrogated the activation of the LUAD cell apoptosis (Fig. 4d). As depicted in Fig. 4e, the let-7b-5p mimic treatment resulted in the invasion defect of LUAD cells, while this deficiency was recovered with DARS2 overexpression (Fig. 4e). Therefore, let-7b-5p downregulation was required to promote DARS2 expression for LUAD progression significantly.

### DARS2 activates PI3K/AKT signaling pathway

**Figure 5:**
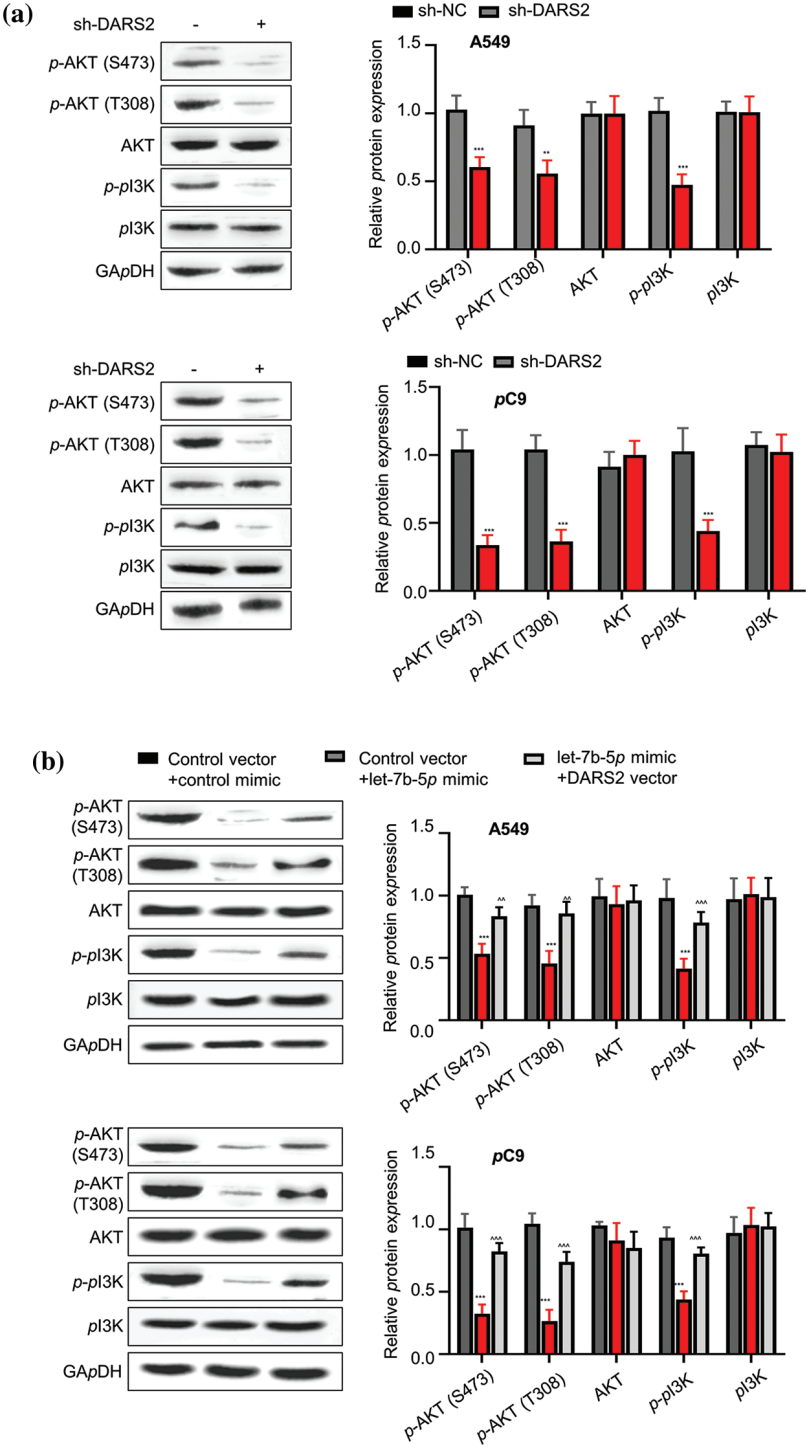
DARS2 activates PI3K/AKT signaling pathway. (a) Western blot analysis presents the expression of p-PI3K,p-AKT, p-AKT, AKT, and PI3K in A549 and PC9 cells transfected with sh-NC and sh-DARS2. (b) Western blot analyzes the expression of p-PI3K,p-AKT, p-AKT, AKT, and PI3K in different groups after transfecting the A549 and PC9 cells with DARS2-overexpressing vectors and let-7b-5p mimic. * represents *p* < 0.05, ** defines *p* < 0.01, and *** indicates *p* < 0.001.

Typically, the hyperactivation of the oncogenic PI3K/AKT signaling pathway characteristically happens to be favorable during tumor progression. Owing to this aspect, we first addressed whether high-expressed DARS2 could lead to LUAD malignancy by PI3K/AKT signaling pathway in A549 and PC9 cells. Due to silencing DARS2, the expression levels of p-PI3K, p-AKT, and p-AKT were constrained (Fig. 5a). As anticipated, the let-7b-5p mimic treatment diminished the expression levels of p-PI3K,p-AKT, and p-AKT proteins. At the same time, the inactivation of the PI3K/AKT signaling pathway was restored by DARS2 overexpression in A549 and PC9 cells (Fig. 5b). Thus, these data suggested that let-7b-5p targeting DARS2 diminished the PI3K/AKT signaling pathway in LUAD cells.

### DARS2 silence constrains LUAD growth in vivo

**Figure 6:**
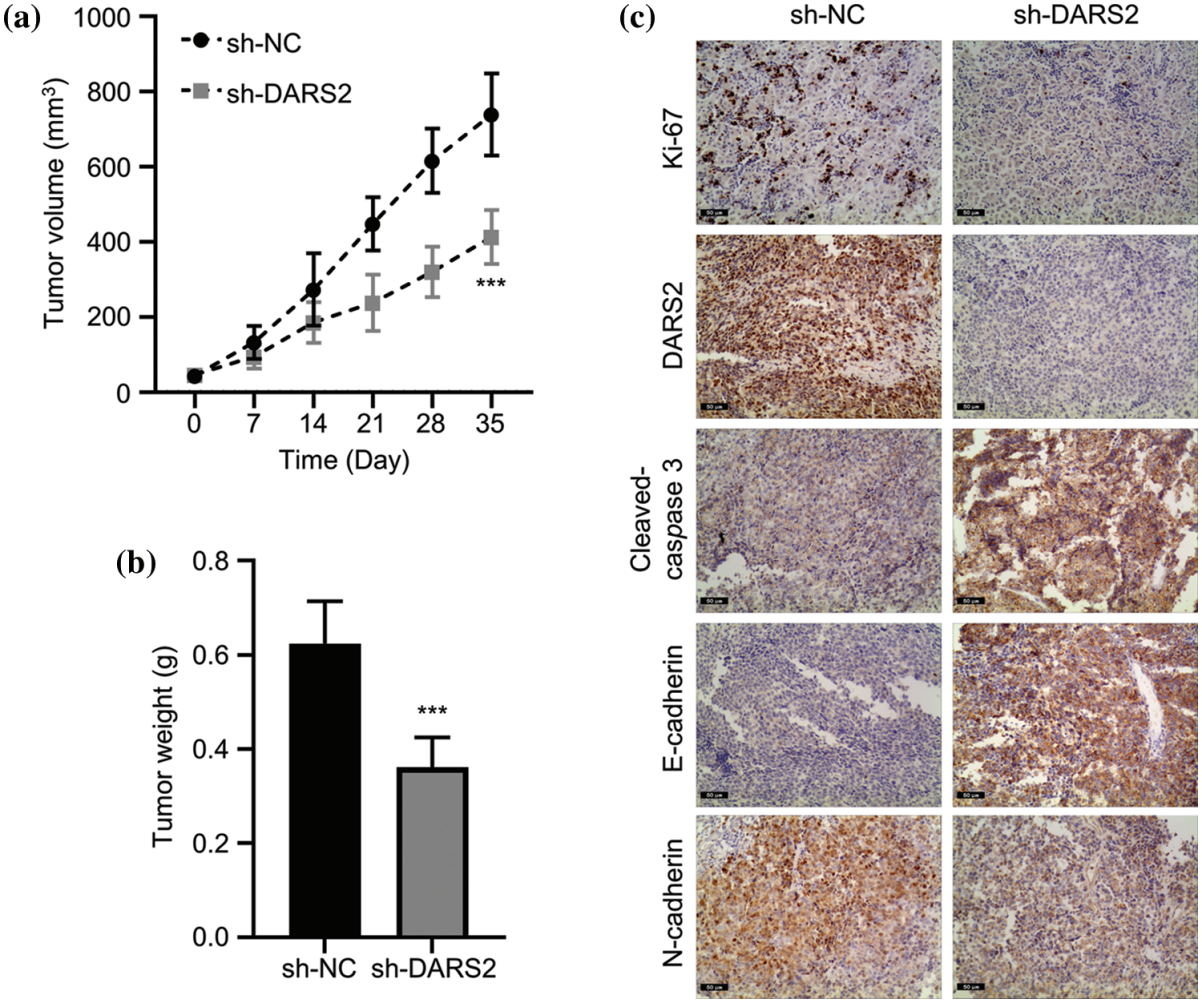
DARS2 silence constrains LUAD growth *in vivo*. (a) The graph shows the tumor volumes of subcutaneously injected mice with A549 cells with sh-NC or sh-DARS2. The data shows (b) tumor weights, and (c) IHC staining analysis of tumor sections with Ki-67, DARS2, cleaved-caspase 3, E-cadherin, N-cadherin,cleaved-caspase 3, and E-cadherin. *** indicates *p* < 0.001.

To validate the role of DARS2 in LUAD progression, a subcutaneous xenograft mouse model was established and explored the findings *in vivo*. Initially, the mouse model was established by subcutaneously implanting A549 cells containing sh-DARS2 or sh-NC in mice. After 35 days, the tumors were resected and weighed. As shown in Figs. 6a and 6b, DARS2 knockdown significantly reduced the volume and weight of the tumors in mice. Significantly, DARS2 knockdown reduced the positive rate of Ki-67, DARS2, and N-cadherin expression levels and increased that of cleaved-caspase 3 and E-cadherin proteins (Fig. 6c). In agreement with the *in vitro* findings, DARS2 silencing substantially restricted the LUAD tumorigenesis *in vivo*.

## Discussion

This study identified a regulatory axis involving let-7b-5p and DARS2 in LUAD cells. The experimental results involving LUAD tissues revealed that DARS2 mRNA was highly expressed and was associated with a poor prognosis in LUAD patients. Further, the loss-of-function assays demonstrated that silencing of DARS2 significantly inhibited the LUAD tumor growth by inactivating the PI3K/AKT signaling pathway. Moreover, this study demonstrated that let-7b-5p could target DARS2, self-downregulating in the LUAD tissues and correlating with a worse prognosis in LUAD patients. Furthermore, it was observed that let-7b-5p overexpression could reverse the proto-oncogenic effects of DARS2 overexpression in LUAD progression. In summary, these findings suggested that targeting the let-7b-5p/DARS2 axis exerted an anti-oncogenic role during LUAD progression by inactivating the PI3K/AKT signaling pathway. In addition, this study provided novel insights into potential intervention strategies for LUAD progression.

Initially, the GEPIA database showed that LUAD tissues displayed increased DARS2 expression and correlated with undesirable clinical outcomes. Moreover, the DARS2 expression and its prognostic value were further subjected to clinical validation. Recently, two bioinformatics analyses underscored the DARS2 overexpression in LUAD patients and correlated to their unfavorable prognosis [15,16]. In a previous report, the upregulation of DARS2 enhanced hepatocarcinogenesis and exerted its oncogenic contribution [6]. Nonetheless, the mechanistic role of DARS2 in the LUAD progression remains unexplored comprehensively. Our data showed that DARS2 silence not only regulated the LUAD cell proliferation, migration, and invasion abilities and triggered apoptosis *in vitro* but also achieved substantial tumor inhibition *in vivo*.

Indeed, the PI3K/AKT signaling pathway plays a crucial role in the development and progression of cancer, regulating several cellular processes, including cell growth, proliferation, survival, metabolism, and angiogenesis, which are crucial for tumor formation and progression [17]. Moreover, the abnormally activated PI3K/AKT signaling pathway significantly contributes to pathological consequences, such as the development of cancers. Notably, the potentiated PI3K phosphorylation is responsible for the phosphorylation of AKT, driving cancer progression [18,19]. Our findings suggested that DARS2 silence reduced the oncogenicity of the PI3K/AKT signaling pathway. Therefore, DARS2 played a promo-oncogenic role during LUAD progression by PI3K/AKT signaling pathway.

Despite the imperfect complementary sequences, gene expression is often reprogrammed by various miRNAs [20]. Using integrative analysis of miRNA profiling, it was observed that let-7b-5p could target DARS2. Previous reports indicated that the aberrantly expressed let-7b-5p was characteristically observed in various cancers [21,22]. Notably, the aberrantly expressed let-7b-5p in various cancers showed context-dependent tumor-promoting and tumor-suppressive effects. For example, let-7b-5p could downregulate tumor protein p53 (p53) and a tumor suppressor transcription factor, leading to the loss of ferroptosis and the suppression of acute myeloid leukemia [23]. In another instance, let-7b-5p overexpression suppressed multiple myeloma cell viability and apoptosis activation [24]. Conversely, let-7b-5p loss remarkably suppressed ovarian cancer cell viability, constraining the characteristics of stem cells [25]. Considerably, our study demonstrated that let-7b-5p expression was downregulated in LUAD tissues, which could be associated with a poor prognosis. Interestingly, ectopic expression of let-7b-5p counteracted the promotion of LUAD cell growth, migration, and invasion abilities by DARS2, offsetting the activation of the PI3K/AKT signaling pathway. Further, the negative correlation between let-7b-5p and DARS2 confirmed the targeted relationship between them. In summary, the downregulation of let-7b-5p could play a crucial role in the DARS2 action, contributing to LUAD progression.

In our study, we have demonstrated that DARS2 downregulation inhibited LUAD cell proliferation, migration, and invasion abilities, substantially retarding tumor growth *in vivo* by inactivating the PI3K/AKT signaling pathway. Moreover, it was demonstrated that let-7b-5p targeted DARS2 expression, counteracting the promotion of LUAD cell proliferation, migration, and invasion abilities. In addition, let-7b-5p attenuated the activated PI3K/AKT signaling pathway by DARS2 overexpression. Therefore, targeting the let-7b-5p-DARS2-PI3K/AKT axis suppressed the LUAD tumor growth and invasion, highlighting a potential therapeutic approach for LUAD treatment. However, this study possessed certain limitations. Firstly, other miRNAs may also target DARS2 due to imperfect complementary recognition of miRNAs to its 3'UTR. To explore this aspect, further studies will be required to elucidate the mechanism of DARS2-driven LUAD progression. Secondly, it was reported that DARS2 regulated the MAPK signaling pathway [6]. In addition, it may regulate a complex and sophisticated gene network to contribute to LUAD progression.

## Data Availability

All data was included in the MS.
